# MicroRNA‐186 is associated with hypoxia‐inducible factor‐1α expression in chronic obstructive pulmonary disease

**DOI:** 10.1002/mgg3.531

**Published:** 2018-12-21

**Authors:** Li Lin, Juan Sun, Duoyi Wu, Daobo Lin, Dingwei Sun, Quanni Li, Jiannan Chen, Huan Niu, Ping He, Yipeng Ding

**Affiliations:** ^1^ Department of Geriatrics the Second Affiliated Hospital of Hainan Medical University Haikou China; ^2^ Department of General Pracitce Hainan General Hospital Haikou China

**Keywords:** chronic obstructive pulmonary disease, HIF-1α, inflammatory cytokines, miR‐186

## Abstract

**Background:**

MicroRNAs (miRNAs) are a family of small noncoding RNAs and are essential in the regulation of gene expression. Their impacts on gene expression have been reported in various diseases. The role of hypoxia‐inducible factor 1 alpha (HIF‐1α) in the development and progression of chronic obstructive pulmonary disease (COPD) has also been demonstrated. However, the role of microRNA‐186 (miR‐186) in relation to HIF in COPD is unknown.

**Methods:**

Cell culture experiments were performed using human lung fibroblast cells (MRC‐5). Cell viability was determined by MTT and flow cytometry assays. Reverse transcriptase‐polymerase chain reaction (RT‐PCR) and Western blot analysis were used to assess the expression levels of HIF‐1α and inflammatory cytokines. Dual‐luciferase reporter assays were used to reveal the correlation between miR‐186 and HIF‐1α.

**Results:**

After miR‐186 transfection, the cell lines showed reduced proliferation and increased apoptosis. After overexpression of miR‐186, we found that the HIF‐1α expression level was reduced in MRC‐5 cells. We found that miR‐186 can affect apoptosis of inflammatory fibroblasts through the regulation of HIF‐1α and affect the downstream signaling pathways.

**Conclusions:**

These data suggested that miR‐186 contributes to the pathogenesis of COPD and that miRNA‐186 may also affect the HIF‐1α‐dependent lung structure maintenance program.

## 1 INTRODUCTION

Chronic obstructive pulmonary disease (COPD) is the fourth leading causes of morbidity and mortality in third world countries and is predicted to become the third leading cause of death in third world countries by the year 2020 (Lópezcampos, Tan, & Soriano, 2016).

Chronic obstructive pulmonary disease is characterized by a progressive and irreversible airflow limitation associated with an abnormal lung inflammatory response to harmful particles and gases (Hansel et al., 2015; Scioscia et al., 2017).

The pathogenesis of COPD remains unclear. MicroRNAs (miRNAs) are a family of small noncoding RNAs, usually 21–25 nucleotides in length, that are essential for the regulation of gene expression (Zou, Liu, Gong, Hu, & Zhang, 2017). miRNAs recognize specific complementary sequences primarily in the 3′‐untranslated region (UTR) of their target mRNAs (Hammond, 2015). miRNAs can regulate cell proliferation, differentiation, apoptosis, and responses to injury or adaptation to chronic stress (Gangaraju & Lin, 2009). It has been reported that the dysregulation of miRNAs is involved in the pathogenesis of many diseases, including pulmonary diseases such as COPD (Barreiro, 2016; Ezzie et al., 2012). Identifying the expression patterns of miRNAs in COPD may enhance our understanding of the disease mechanisms.

MicroRNA‐186 (miR‐186) has been shown to be one of the most important determinants of cell proliferation in various types of cancers (Cai et al., 2013). The miRNAs hsa‐miR‐146b, hsa‐miR‐141, hsa‐miR‐186, and hsa‐miR‐196b‐5p were differentially expressed in our previous study (Ding et al., 2016). As far as we know, chronic hypoxia is a common feature of COPD. Physiological responses to chronic hypoxia result from altered patterns of gene expression. An essential regulator of the response to decreased O_2_ is hypoxia‐inducible factor‐1 (HIF‐1) (Shimoda & Semenza, 2011). Recent data indicated that HIF‐1α plays a major role in COPD (Putra, Tanimoto, Arifin, Antariksa, & Hiyama, 2013). The role of miR‐186 in the pathogenesis of COPD via regulation of HIF‐1α is unclear. Therefore, in our study, we used in vitro experiments to determine whether and how the miR‐186/HIF‐1α axis influences human embryonic lung fibroblasts and whether it has role in COPD.

## 2 MATERIALS AND METHODS

### 2.1 Cell culture and transfection

The MRC‐5 human lung fibroblast cell lines were purchased from the Chinese Academy of Sciences Shanghai Branch (Shanghai, China). MRC‐5 cells were grown in Dulbecco’s Modified Eagle’s Medium (DMEM; Thermo Fisher Scientific, Inc., Waltham, MA, USA) supplemented with 10% fetal bovine serum (FBS; Sigma‐Aldrich, St. Louis, MO, USA) and 100 units/ml Invitrogen penicillin–streptomycin (Thermo Fisher Scientific, Inc.). The cells were cultured in a humidified incubator at 37˚C in 5% CO_2_ and 95% air atmospheres for 2–3 days. miRNA transfection was performed using Lipofectamine 2000 in accordance with the manufacturer's guidelines (Invitrogen). Untreated cells were designated as the control group.

### 2.2 Cell viability assays

Cell viability was determined using the 3‐(4, 5)‐dimethylthiahiazo (−z‐y1)‐3,5‐di‐phenytetrazoliumromide (MTT; Beyotime, Jiangsu, China) assay. Briefly, 2.0 × 10^3^ cells/well were seeded in a 96‐well plate and allowed to adhere. At different time points, 20 μl of 5 mg/ml MTT solution was added to each well of the plate, and the plates were incubated for 4 hr. Next, the liquid was removed from the plate and 100 μl of DMSO was added to the wells, the mixture was agitated for 5 min, and the OD was measured at 490 nm.

Flow Cytometry: Cells were harvested at the indicated time points, washed, and labeled with allophycocyanin–annexin V or FITC–annexin V (eBioscience) and propidium iodide (Invitrogen), or 7‐aminoactinomycin D (eBioscience). Flow cytometric analysis was carried out on a MACSQuant analyzer (Miltenyi Biotec) and analyzed using FlowJo software (Tree Star).

### 2.3 Real‐time reverse transcriptase–polymerase chain reaction

Total RNA was extracted from MRC‐5 cells using TRIzol®. qPCR was performed with 2 μg of total RNA using AMV reverse transcriptase and random primers. The PCR primers were designed according to the sequences in GenBank. cDNA amplification was performed according to the manufacturer’s protocol using a SYBR Premix Ex Taq II kit (Takara, Kyoto, Japan). GAPDH mRNA levels were measured for normalization.

### 2.4 Protein isolation and Western blot analysis

MRC‐5 cells were lysed, the proteins were extracted, and Western blotting was performed (Towbin & Staehlin, 1979; Towbin, Staehelin, & Gordon, 1979). The immunoblots were washed with Western blot stripping buffer (pH 2–3; Nacalai, Tokyo, Japan) and probed with monoclonal antibodies against GAPDH (1:2,000; Proteintech Group, Chicago, IL, USA).

### 2.5 Dual‐luciferase reporter assay

The pGL3‐luciferase reporter gene plasmid pGL3‐ 3′‐UTR (GeneCopoeia, Guangzhou, China) was cotransfected into the cells with 15 mol of the miR‐186 mimic, miR‐186 control, or miR‐186‐mut and 5 ng pRL‐TK *Renilla *plasmid (Promega Corporation, , Madison, WI, USA) using Invitrogen Lipofectamine 2000 reagent. The cells were collected 48 hr after transfection and analyzed using the dual‐luciferase reporter assay system (Promega). The detected luciferase activity was normalized to the activity of *Renilla* luciferase. Each reporter plasmid was transfected at least three times, and each sample was assayed in triplicate.

### 2.6 Statistical analyses

All data are presented as the mean ± standard deviation (*SD*) of the indicated number of observations. Paired or unpaired *t* tests (two‐tailed) were used as a parametric method for testing statistical significance. Correlation analysis of normally distributed data was carried out using Pearson’s one‐tailed correlation test reflecting the inverse relation of the expression of miRNAs and their associated targets. A *p*‐value <0.05 was considered to be statistically significant.

## 3 RESULTS

### 3.1 miR‐186 promoted MRC‐5 cell proliferation and apoptosis

**Figure 1:**
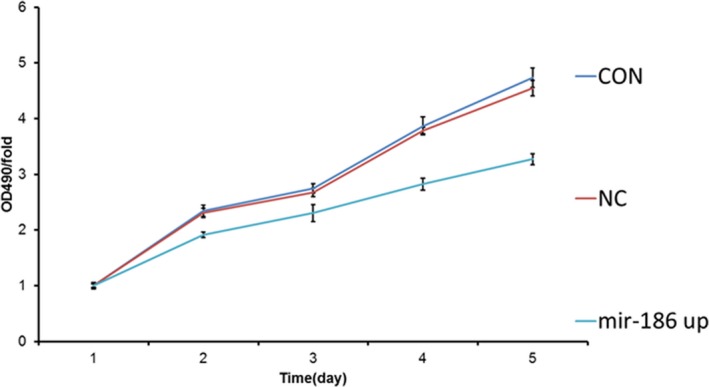
The effects of microRNA‐186 (miR‐186) on the viability of MRC‐5 cells. The viability of the MRC‐5 cells was measured using MTT

**Figure 2:**
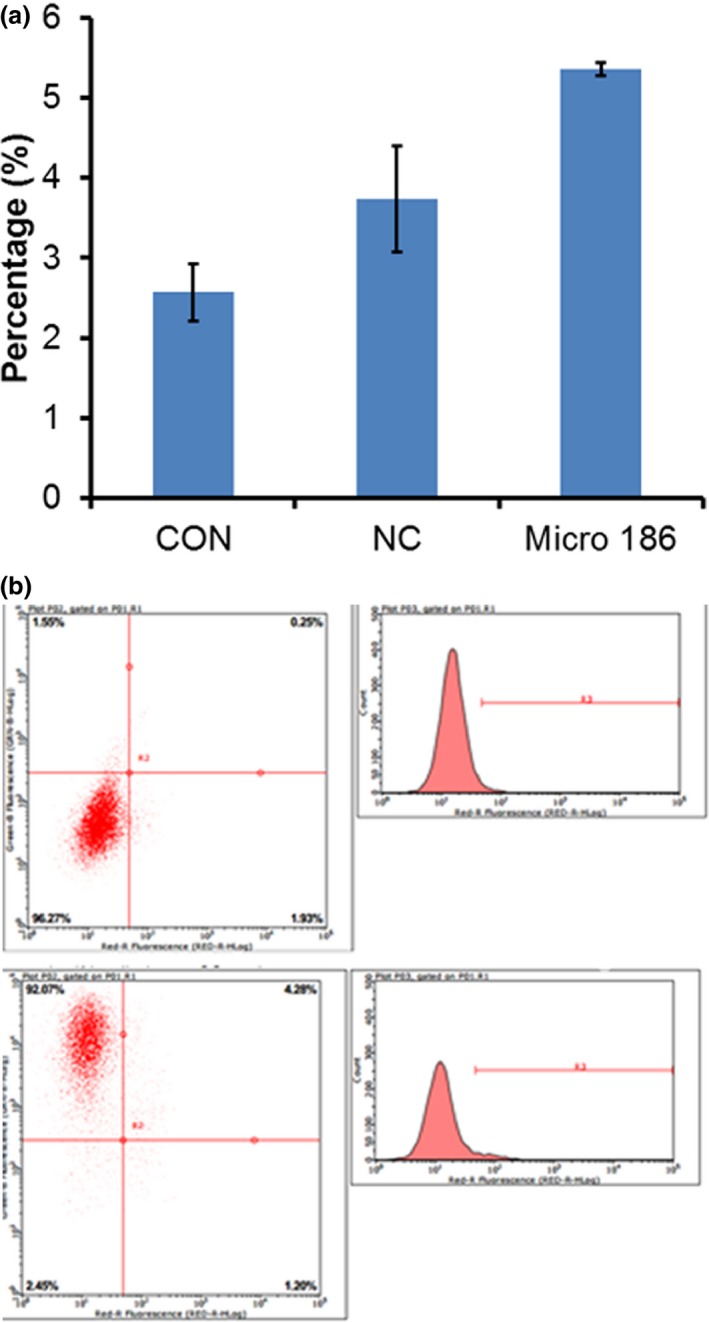
(a) The effects of microRNA‐186 (miR‐186) on apoptosis in MRC‐5 cells. (b) Assessment of apoptosis using flow cytometry with Annexin V‐FITC/PI‐staining

To explore the effects of miR‐186 on the growth of MRC‐5 cells, MRC‐5 cells were transfected with miR‐186 mimics. The results of the MTT assays showed that miR‐186 significantly inhibited the proliferation of MRC‐5 cells (Figure 1); furthermore, we investigated the role of miR‐186 in cell apoptosis. Our results show that miR‐186 overexpression could induce apoptosis, while NC‐transfected MRC‐5 cells showed no effect (Figure 2).

**Figure 3:**
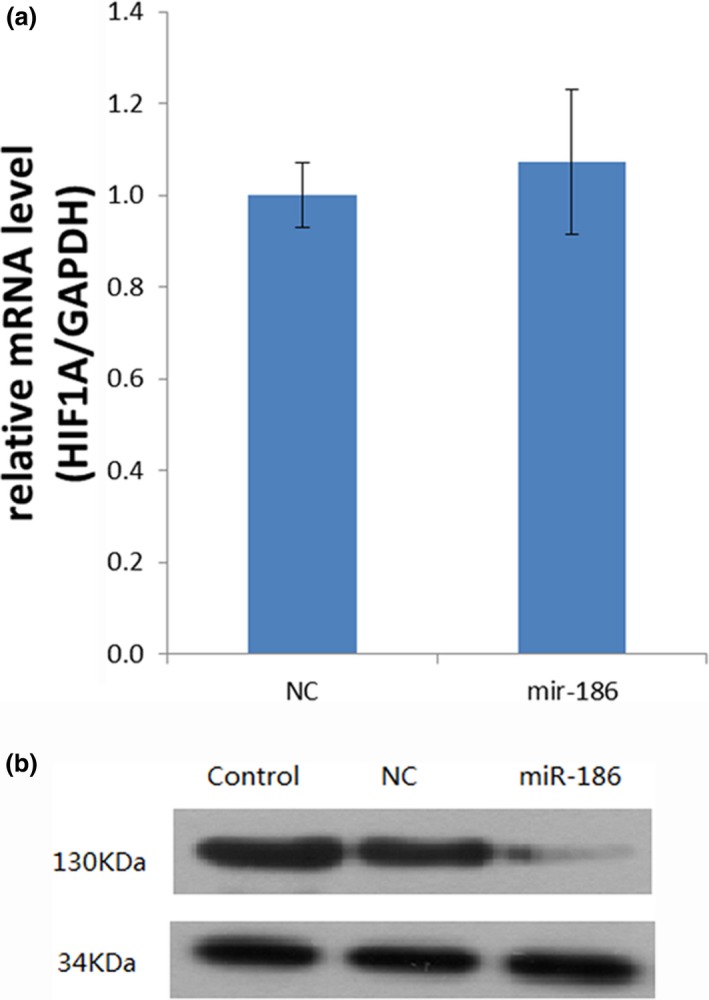
(a) Reverse transcriptase–polymerase chain reaction for HIF‐1α in MRC‐5 cells. (b) Western blots for HIF‐1α in MRC‐5 cells

We next transfected miR‐186 into MRC‐5 cells and analyzed the mRNA and proteins levels of HIF‐1α. miR‐186 overexpression downregulated the expression of HIF‐1α. miR‐186 overexpression inhibited the expression levels of HIF‐1α mRNA in MRC‐5 cells, while anti‐miR‐186 transfection upregulated HIF‐1α expression compared with negative‐control cells or mock‐transfected cells (Figure 3a). Similarly, the results of the Western blot analysis revealed that the HIF‐1α protein levels were decreased after miR‐186 transfection but remained higher than the corresponding levels in the negative‐control and mock‐transfected groups (Figure 3b). Taken together, the above results clearly demonstrated that miR‐186 regulates HIF‐1α by directly targeting the HIF‐1α mRNA in vivo in MRC‐5 cells.

### 3.2 Bioinformatics and luciferase reporter assay

**Figure 4:**
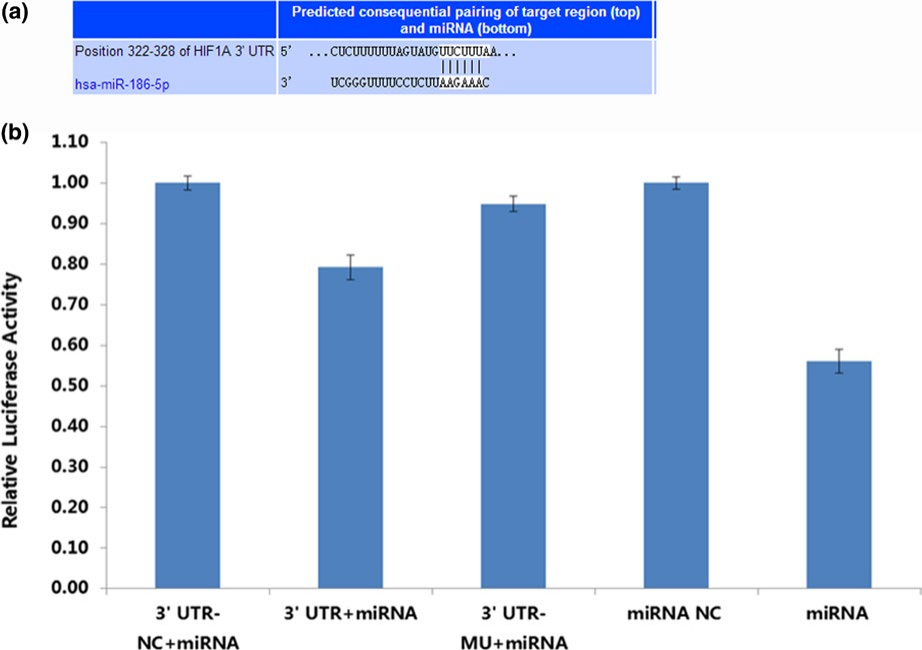
(a) The sequence of the 3′‐untranslated region (3′‐UTR) of the HIF‐1α mRNA and the microRNA‐186 (miR‐186) binding site. (b) Bioinformatics and luciferase reporter assay results. MiR‐186 significantly decreased the relative luciferase activity of the wild‐type HIF‐1α 3‐UTR compared with the mutant HIF‐1α 3′‐UTR

Computational programs predicted that the 3′‐UTR of HIF‐1α contains a potential miRNA‐binding site for miR‐186 (Figure 4a). To confirm that HIF‐1α is a bona fide target of miR186, we performed a luciferase reporter assay using sequences from the wild‐type or mutant 3′‐UTR of HIF‐1α. The relative luciferase activity of the wild‐type HIF‐1α 3′‐UTR was significantly downregulated by miR‐186 compared with the mutant HIF‐1α 3′‐UTR, thereby indicating that miR‐186 may directly bind to the 3′‐UTR of HIF‐1α (Figure 4b).

## 4 DISCUSSION

The main finding of this work is that therole of miR‐186 in the pathogenesis of COPD is the regulation of HIF‐1α expression. Previously, we used miRNA‐ChIP and reverse transcriptase‐polymerase chain reaction (RT‐PCR) to assess the distribution and differences in miRNA expression in the peripheral blood of COPD patients in comparison to normal subjects. The results showed 13 miRNAs were differentially expressed between COPD patients and the control subjects. Further research using RT‐PCR confirmed that the expression levels of the following seven miRNAs were significantly different: hsa‐miR‐146b‐5p, hsa‐miR‐141‐3p, hsa‐miR‐186‐5p, hsa‐miR‐4532, hsa‐miR‐4635, hsa‐miR‐2681‐3p, hsa‐miR‐4503, and hsa‐miR‐196b‐5p (Ding et al., 2016). To investigate whether these miRNAs are associated with COPD, we conducted further experiments in which miRNAs were transfected into human lung fibroblasts. The results showed that in comparison with control cells, human lung fibroblasts transfected with miR‐146b‐5p and miR‐141‐3p had decreased apoptosis and increased proliferation, and cells transfected with hsa‐miR‐186 had increased apoptosis and decreased proliferation. Cells transfected with miR‐196b‐5p showed no significant phenotypes. According to the results, miR‐146b and miR‐141 upregulation accelerated MRC‐5 cell proliferation and decreased apoptosis, and miR‐186 upregulation slowed MRC‐5 cell proliferation and increased apoptosis. The imbalance of apoptosis and proliferation in pulmonary fibroblasts is an important factor in the reconstruction of COPD airways (Zheng‐Xing, Bo, & Zhou, 2013); therefore, regulating the function of fibroblasts, including inducing apoptosis and inhibiting their proliferation, are targets of COPD treatment. Based on these previous observations, we chose miR‐186 for further study. MicroRNA‐186 (miR‐186) has turned out to be one of the most important determinants of cell proliferation in various types of cancers (Cai et al., 2013; Ruan, Xiaoting, Jun, & Hang, 2016; Zhang et al., 2010). COPD is a chronic systemic inflammatory syndrome characterized by irreversible, progressive airflow obstruction (Thorleifsson et al., 2009). HIF‐1α is a critical factor responsible for maintaining oxygen homeostasis under hypoxic conditions, and this transcription factor is thought to be associated with the development and progression of COPD (Putra et al., 2013). In recent years, HIF‐1α has emerged as a key factor in COPD. HIF‐1 also plays a major role in the expression of genes involved in inflammation, energy metabolism, angiogenesis, and airway remodeling (Balamurugan, 2016). The role of HIF in the development and progression of COPD has also been demonstrated. For instance, previous studies showed that HIF‐1 plays an important role in critical pathological processes of pulmonary heart disease in patients with hypoxic pulmonary hypertension and COPD (Fan et al., 2011; Manalo et al., 2005; Qian, Wang, Ming, & Liang, 2012; Schultz, Fanburg, & Beasley, 2006; Xie et al., 2017). Our results showed that mir‐186 has a binding site in the 3′‐UTR of HIF‐1α. We found that mir‐186 can affect apoptosis of inflammatory fibroblasts via regulation of HIF‐1α and affect the downstream signaling pathways.

In our study, the roles of miR‐186, HIF‐1α and their inflammatory cytokines in COPD pathogenesis contributes to the search for new signaling pathways involved in COPD development. These signaling pathways may become a new, useful, and potential target for clinical treatment and diagnosis, providing new means and assistance for the treatment of COPD.

## CONFLICT OF INTEREST

None declared.
